# Rugby World Cup 2019 injury surveillance study

**DOI:** 10.17159/2078-516X/2020/v32i1a8062

**Published:** 2020-01-01

**Authors:** CW Fuller, A Taylor, M Douglas, M Raftery

**Affiliations:** 1 Colin Fuller Consultancy Ltd, Sutton Bonington, United Kingdom; 2 World Rugby, World Rugby House, 8–10 Pembroke Street Lower, Dublin 2, Ireland

**Keywords:** Rugby World Cup, injury incidence, injury severity, injury burden, injury risk

## Abstract

**Background:**

Full contact team sports, such as rugby union, have high incidences of injury. Injury surveillance studies underpin player welfare programmes in rugby union.

**Objective:**

To determine the incidence, severity, nature and causes of injuries sustained during the Rugby World Cup 2019.

**Methods:**

A prospective, whole population study following the definitions and procedures recommended in the consensus statement for epidemiologic studies in rugby union. Output measures included players’ age (years), stature (cm), body mass (kg), playing position, and group-level incidence (injuries/1000 player-hours), severity (days-absence), injury burden (days absence/1000 player-hours), location (%), type (%) and inciting event (%) of injuries.

**Results:**

Overall incidences of injury were 79.4 match injuries/1000 player-match-hours (95% CI: 67.4 to 93.6) and 1.5 training injuries/1000 player-training-hours (95% CI: 1.0 to 2.3). The overall mean severity of injury was 28.9 (95% CI: 20.0 to 37.8) days absence during matches and 14.8 (95% CI: 4.1 to 25.5) days absence during training. The most common locations and types of match injuries were head/face (22.4%), posterior thigh (12.6%), ligament sprain (21.7%) and muscle strain (20.3%); the ankle (24.0%), posterior thigh (16.0%), muscle strain (44.0%) and ligament sprain (16.0%) were the most common locations and types of injuries during training. Tackling (28.7%), collisions (16.9%) and running (16.9%) were responsible for most match injuries and non-contact (36.0%) and contact (32.0%) rugby skills activities for training injuries.

**Conclusion:**

The incidence, severity, nature and inciting events associated with match and training injuries at Rugby World Cup 2019 were similar to those reported for Rugby World Cups 2007, 2011 and 2015.

Athletes competing in contact team sports, such as rugby union, generally experience higher incidences of injury than those competing in non-contact and semi-contact team sports. World Rugby, as the international governing body for rugby union, addresses its duty of care by implementing an evidence-based player welfare strategy underpinned by risk management principles.^[1–3]^ Central to this player welfare strategy is a comprehensive programme of injury surveillance studies implemented at all major men’s and women’s international rugby tournaments.

World Rugby’s flagship competition is the men’s Rugby World Cup (RWC) which is contested every 4 years. The main objectives of the present study were to preserve the World Rugby’s programme of injury surveillance studies for the RWC and to compare the RWC 2019 match injury results with those previously published for the 2007, 2011 and 2015 RWCs respectively.

## Methods

### Study definitions and procedures

The definitions and procedures used in this study were compliant with the consensus statement on injury definitions and data collection procedures for studies of injuries in rugby union^[4]^ and were also consistent with the procedures used for RWCs 2007, 2011 and 2015 studies.^[5–7]^ World Rugby’s Institutional Ethics Committee approved the study and all players taking part in RWC 2019 consented to their data being included in this study. The study took place in Japan over a 7 week period commencing on Monday 16 September 2019. The first match was played on Friday 20 September and the final match on Saturday 2 November 2019. Three scheduled RWC 2019 group stage matches were cancelled due to adverse weather conditions.

Six weeks prior to the start of the competition, the 20 participating countries received a guidance document explaining the definitions and reporting procedures. Each player’s baseline information, consisting of their normal playing position, date of birth, stature (cm) and body mass (kg), was obtained with data reported as population means (standard deviation). Equivalent anthropometric data for players competing in the 2007, 2011 and 2015 RWCs were retrieved from previous publications.^[5–7]^ Match exposures were based on 15 players (backs: 7; forwards: 8) being exposed for 80 minutes per match. No allowances were made for players temporarily (as a result of medical treatment) or permanently (due to the receipt of yellow or red cards) missing during a match. None of the matches required extra time. Training exposures were recorded based on the number of players (backs, forwards) attending team training sessions, and the number, length (in minutes) and structure of the sessions (preparation: warm up, cooldown, rugby skills: full-contact, semi-contact, non-contact, conditioning: with weights, without weights; other activities).

For the RWC, the definition of an injury was: ‘Any physical complaint sustained by a player during a RWC match or training session that prevented the player from taking a full part in all training activities or match play for more than 1 day following the day of injury, irrespective of whether match or training sessions were actually scheduled*’*.^[4]^ The definition of an illness was: *‘*Any medical condition sustained during the period of the RWC study that prevented the player from taking a full part in all training activities and/or match play for more than 1 day following the day of onset of the illness.*’*. Injuries were reported as recurrences based on the clinical judgement of the injured player’s medical team using the definition: ‘An injury of the same type and at the same site as an index injury and which occurred after the player’s return to full participation from the index injury’.^[4]^ Team physicians/physiotherapists were responsible for reporting injuries/illnesses, including the date of injury/illness, the date of return-to-play/training, injury location, type, Orchard Sports Injury Coding System (v8) code,^[8]^ and recurrence. All concussion injury reports were cross-checked for consistency with the results reported for match day concussion assessments. When necessary, injuries and illnesses were followed up for three months after the final match of RWC 2019 to obtain return-to-play/training dates. Beyond this time, team physicians provided estimated return-to-play/training dates based on a player’s condition at that time (eight injuries). Where appropriate, risk factors, such as playing position, time of match injury (0 – 20; 21 – 40+; 41 – 60; 61 – 80+ minutes), activity at time of injury and whether the player was removed from play/training when injured, were reported. In this study, match and training results are reported separately as the number of injuries/1 000 player-hours of exposure with 95% confidence intervals (CI) and injury severities are reported as the mean and median (days; 95% CI). Injury burden, the product of incidence and mean severity of injury,^[2,9,10]^ is reported as days-absence/1 000 player-hours (95% CI). Equivalent injury data for the 2007, 2011 and 2015 RWCs were retrieved from other published reports.^[5–7]^

### Statistical analysis

Statistical comparisons of players’ anthropometric data were calculated using unpaired t-tests.^[11]^ Numbers of injuries were compared using chi-squared tests, z-tests were used to compare incidences and mean severities of injury, and Mann-Whitney U-tests for median severities.^[11]^ Trends in values over the four RWCs (2007, 2011, 2015, 2019) were calculated using linear regression analyses and are reported with the slope of the regression lines and R^2^ values.^[11]^ The number of data comparisons made in this study create the potential for some comparisons to appear as statistically significant at the p≤0.05 level when they may have occurred by chance; for this reason, exact p-values are reported for statistical tests.

## Results

Six hundred and forty-six players (backs: 284; forwards: 362) representing 20 countries took part in this study and provided baseline anthropometric data (Table 1).

There were no significant trends in players’ age (backs: slope = 0.024 years/year, R^2^ = 0.12, p = 0.653; forwards: slope = −0.051 years/year, R^2^ = 0.46, p = 0.332), stature (backs: slope = 0.018 cm/year, R^2^ = 0.31, p = 0.443; forwards: slope = −0.068 cm/year, R^2^ = 0.72, p = 0.151) or body mass (backs: slope = −0.044 kg/year, R^2^ = 0.08, p = 0.726; forwards: slope = 0.10 kg/year, R^2^ = 0.50, p = 0.296) over the period 2007 to 2019.

### Incidence, severity and injury burden

One hundred and forty-three match injuries (backs: 79; forwards: 64) were sustained during the 45 matches played (group stage: 37; knockout stage: 8), which equate to 1 800 player-match-hours (backs: 840; forwards: 960). Over the 7 week period, a total of 16 220 (backs: 7 168; forwards: 9 052) player-training-hours (3 teams did not return player-training-hours) and 25 training injuries (backs: 11; forwards: 14) (all teams returned training injuries) were recorded: incidence of training injuries was based on the results provided by the 17 teams returning both injury and exposure data. No catastrophic spinal or career-ending injuries were reported during the tournament. Two illnesses were reported during the tournament (tonsillitis: 1; ear infection: 1). The illnesses correspond to a period prevalence of 0.3% over the period of the competition. No further analysis of these illnesses was undertaken due to their small number.

The incidences and severities of match injuries sustained at RWC 2019 are presented in Table 2. It shows that the incidence of injury was higher for backs than forwards (difference = 27.4 injuries/1 000 player-match-hours, 95% CI = 1.0 to 53.8, p = 0.041). There were no trends in the incidences of match injuries over the four RWCs for backs (average value = 93.0 injuries/1 000 player-match-hours, slope = 0.94 injuries/1 000 player-match-hours/year, R^2^ = 0.49, p = 0.299) or forwards (average value = 79.2 injuries/1 000 player-match-hours, slope = −1.4 injuries/1 000 player-match-hours/year, R^2^ = 0.72, p = 0.151). There was no significant difference between the severities of match injuries sustained by backs and forwards at RWC 2019 (mean severity: difference = −0.9 days, 95% CI = −19.0 to 17.2, p = 0.920; median severity: difference = −1.0 days, p = 0.992). There was no trend in the severity of match injuries over the four RWCs for backs (mean severity: slope = 1.1 days/year, R^2^ = 0.70, p = 0.162; median severity: slope = 0.00 days/year, R^2^ <0.01, p = 1.000) but an increasing trend for forwards (mean severity: slope = 1.4 days/year, R^2^ = 0.91, p = 0.049; median severity: slope = 0.25 days/year, R^2^ = 0.83, p = 0.087).

The incidences and severities of training injuries sustained at RWC 2019 are also included in Table 2. The incidence of training injuries was higher for forwards than backs (difference = 0.26 injuries/1 000 player-training-hours, 95% CI = −0.9 to 1.5, p = 0.674). There were no significant trends in the incidence of training injuries over the four RWCs from 2007 to 2019 for either backs (average value = 1.9 injuries/1 000 player-training-hours, slope = 0.19 injuries/1 000 player-training-hours/year, R^2^ = 0.65, p = 0.192) or forwards (average value = 2.3 injuries/1 000 player-training-hours, slope = −0.17 injuries/1 000 player-training-hours/year, R^2^ = 0.74, p = 0.141). There was no significant difference between the severities of training injuries sustained by backs and forwards (mean severity: difference = 3.7 days, 95% CI = −22.5 to 29.9, p = 0.779; median severity: difference = −1.0 day, p = 0.978). Also, there was no trend in the mean severity (slope = −0.22 days/year, R^2^ = 0.03, p = 0.825) but a decreasing trend in the median severity (slope = −0.38 days/year, R^2^ =1.00, p <0.001) of training injuries sustained by backs over the period 2007 to 2019. There were no trends for forwards in the mean (slope = −0.54 days/year, R^2^ = 0.10, p = 0.691) or median (slope = −0.10 days/year, R^2^ = 0.20, p = 0.553) severities.

In total, 4 526 player-days were lost as a consequence of match (backs: 2 251; forwards: 1 879) and training (backs: 196; forwards: 200) injuries sustained in the RWC 2019. The overall match injury burden experienced was 2 296 days absence/1 000 player-match-hours (95% CI = 1 949 to 2 705); with the injury burden for backs (2 680 days absence/1 000 player-match-hours, 95% CI = 2 115 to 3 342) higher than that for forwards (1 957, 95% CI = 1 532 to 2 501, p = 0.061). There were no statistically significant trends in injury burden for backs (slope = 118 days absence/1 000 player-match-hours/year, R^2^ = 0.65, p = 0.192) or forwards (slope = 73 days absence/1 000 player-match-hours/year, R^2^ = 0.58, p = 0.236) over the period 2007 to 2019. The overall training injury burden for RWC 2019 was 23 days absence/1 000 player-training-hours (95% CI = 15 to 34). The training injury burden for backs was similar (24 days absence/1 000 player-training-hours, 95% CI = 15 to 34) to that for forwards (22, 95% CI = 13 to 37, p = 0.865).

### Location, type and nature of injuries

Ninety-one per cent (95% CI = 87.0 to 96.1) of match injuries were acute (backs: 92.3%, 95% CI = 86.4 to 98.2; forwards: 90.6%, 95% CI = 83.5 to 97.8; p=0.719) and 8.5% (95% CI = 3.9 to 13.0) gradual onset (backs: 7.7%, 95% CI = 1.8 to 13.6; forwards: 9.4%, 95% CI = 2.2 to 16.5; p = 0.719). Of the 25 training injuries, 84.0% (95% CI = 69.6 to 98.4) were acute (backs: 70.0%, 95% CI = 41.6 to 98.4; forwards: 93.3%, 95% CI = 80.7 to 100; p = 0.119) and 16.0% (95% CI: 1.6 to 30.4) gradual onset (backs: 30.0%, 95% CI = 1.6 to 58.4; forwards: 6.7%, 95% CI = 0 to 19.3; p = 0.119).

The proportions of match injuries sustained as functions of location and type of injury are presented in Tables 3 and 4. There were no statistically significant trends in the proportions of the main injury locations or injury types sustained over the period 2007 to 2019 for backs or forwards, apart from an increasing trend in the proportion of head/neck injuries sustained by backs (slope = 1.6%/year, R^2^ = 0.95, p = 0.028).

The six most common match injuries and the six match injuries responsible for most days absence are presented in Table 5. The knee ligament group of injuries includes medial (5), anterior cruciate (1), posterior cruciate (1) and complex (1) ligament injuries.

As a consequence of their match injuries, 31.0% (95% CI: 23.4 to 49.1) of players (backs: 34.6%, 95% CI 24.1 to 45.2; forwards: 26.6%, 95% CI 15.7 to 37.4; p = 0.303) were removed from play immediately, 20.4% (95% CI 13.8 to 36.2) of players (backs: 15.4%, 95% CI 7.4 to 23.4; forwards: 26.6%, 95% CI 15.7 to 37.4; p = 0.101) were removed later in the game and 48.6% (95% CI 40.4 to 68.2) of players (backs: 50.0%, 95% CI 38.9 to 61.1; forwards: 46.9%, 95% CI 34.6 to 59.1; p = 0.711) remained on the pitch until the end of the game. Of the 22 match concussions reported, 17 players (77.3%) were removed from play immediately, three (13.6%) players were removed later in the game and two (9.1%) players completed the game.

Seventy-six per cent of training injuries (ankle: 24.0%, 95% CI = 7.3 to 40.7; posterior thigh: 16.0%, 95% CI = 1.6 to 30.4) were lower limb injuries and sixty-four per cent were muscle/tendon or joint (non-bone) injuries (muscle strains: 44.0%, 95% CI = 24.5 to 63.5; ligament sprains: 16.0%, 95% CI = 1.6 to 30.4). One training injury was a concussion which was identified during the training session and the player was removed immediately. A more detailed analysis of the training injuries was not undertaken due to the small number (n=25) reported.

### Risk factors for match and training injuries

There were no significant differences in the anthropometric characteristics of injured players compared to the overall sample of players for **age** (backs: difference = 0.3 years, 95% CI = −0.5 to 1.1 years, p = 0.478; forwards: difference = 0.1 years, 95% CI = −0.8 to 1.0, p = 0.834), **stature** (backs: difference = +0.7 cm, 95% CI = −0.8 to +2.2, p = 0.347; forwards: difference = −1.2 cm, 95% CI = −2.7 to 0.5, p = 0.197) or **body mass** (backs: difference = 0.75, 95% CI = −1.5 to 3.1, p = 0.478; forwards: difference = 0.0, 95% CI = −2.1 to 2.1, p = 1.000). Backs sustained a higher proportion of injuries in the second half of matches compared to the first half but there was little difference for forwards (backs: first half = 37.0%, 95% CI = 25.9 to 48.1, second half = 63.0%, 95% CI = 51.9 to 74.1, p=0.026; forwards: first half = 47.5%, 95% CI = 34.7 to 60.2, second half = 52.5%, 95% CI = 39.8 to 65.3, p=0.696).

Contact events were responsible for the majority of match injuries (contact: 76.0%, 95% CI: 69.6 to 82.4; non-contact: 24.0%, 95% CI: 17.6 to 30.4) and training injuries (contact: 70.0%, 95%CI: 49.9 to 90.1; non-contact: 30.0%, 95% CI: 9.9 to 50.1). The specific activities associated with the match injuries are shown in Table 6.

There were no statistically significant trends in the proportion of injuries sustained for any of the match activities in RWC games over the period 2007 to 2019 for backs or forwards, apart from an increasing trend in the proportion of tackling injuries sustained by backs (slope = 1.7%/year, R^2^ = 0.95, p = 0.028).

## Discussion

Consistent with previous RWC studies,^[5–7]^ forwards were significantly taller and heavier than backs in RWC 2019 but there was no difference in the age of backs and forwards. There was no evidence of significant trends in the age, stature or body mass of backs or forwards over the period RWC 2007 to RWC 2019. The stature and body mass of RWC players over this 12 year period were similar to values reported in previous studies of elite Northern and Southern hemisphere players.^[12,13]^ Although backs and forwards taking part in the RWC were on average two to three years older than players competing at the elite club level,^[12]^ this is to be expected as the more experienced players are generally selected for the national teams. The incidence of match injury for backs was higher than that recorded for forwards in RWC 2019, which has also been reported in previous RWCs.^[5–7]^ The lower overall incidence of match injuries recorded in RWC 2019 compared to RWC 2015 is welcome with the reduction mainly attributable to the lower incidence of injuries recorded by forwards. Although the incidences of training injuries for both backs and forwards were higher during RWC 2019, compared with RWC 2015, the long-term downward trend in the incidence of training injuries remains.

There were no significant differences between the severities of match injuries sustained by backs and forwards during RWC 2019. The increasing trends in mean severity of match injuries reported previously for backs and forwards in the period RWC 2007 to RWC 2015,^[7]^ did not continue in RWC 2019; however, mean severity values remain at the higher end of the previous range. Median severity values have changed little for backs and forwards over the RWC 2007 to RWC 2019 period, which indicates that the main reason for the increase in mean severity continues to be related to small increases in the number of high severity injuries.

The overall lower match injury incidence recorded in RWC 2019, compared to RWC 2015, resulted in a decrease in players’ match injury burden (RWC 2019: 4 130 player-days-absence; RWC 2015: 5 152 player-days-absence);^[7]^ however, player-days-absence in RWC 2019 remained above those reported for RWC 2011 (4 047 player-days-absence) and RWC 2007 (2 366 player-days-absence).^[6,7]^ The injury burden from training injuries sustained in RWC 2019 (396 player-days-absence) remained similar to that reported for RWC 2015 (287 player-days-absence) but were much lower than those reported for RWC 2007 (1 065 player-days-absence) and RWC 2011 (940 player-days-absence).^[6,7]^

The head/face (21.9%), posterior thigh (10.9%) and knee (15.6%) are the sites of almost 50% of all injuries sustained, whilst ligament sprains (21.7%), muscle strains (20.3%) and concussions (15.4%) describe almost 60% of all injury types sustained. While the two most common specific injuries (concussion: 15.4%; hamstring muscle strain: 9.8%) accounted for approximately 25% of all injuries sustained in RWC 2019, they were not responsible for the most time-loss injuries, as knee ligament (22.6%) and knee cartilage (12.0%) injuries together accounted for nearly 35% of all time lost. Although there was a small increase in match concussions in RWC 2019 compared to RWC 2015 (15.5% v 13.9%; p = 0.582), when expressed as a percentage of all match injuries sustained, the incidence of concussion was slightly lower in RWC 2019 than RWC 2015 (12.2 v 12.5 injuries/1 000 player-match-hours; p = 0.912) due to the overall reduction in injury incidence in RWC 2019. A comparison of concussions sustained in the 2007, 2011 and 2015 RWCs has been presented previously.^[7]^. Despite concussions currently being the focus for injury prevention in rugby, knee ligament, knee cartilage and hamstring injuries together are responsible for nearly 50% of all player-days-absence. These injuries, therefore, must not be overlooked, as they are likely to provide a greater return on any investment in injury prevention.^[14]^

The tackle (tackling: 28.7%; being tackled: 19.1%) was responsible for 47.8% of all injuries sustained in RWC 2019, which continues the upward trend in the proportion of injuries sustained in the tackle observed in previous RWCs (2007: 29.2%; 2011: 40.1%; 2015: 45.9%)^[5–7]^. A number of previous studies have investigated relationships between match injuries and specific match activities^[15–18]^.

This study has a number of strengths: it is a prospective, whole population study following the recommendations of the consensus statement on injury definitions and data collection procedures for studies of injuries in rugby union.^[4]^ The study describes the sample populations in terms of players’ position, age, stature and body mass. Injuries and illnesses were diagnosed and reported by qualified team doctors and physiotherapists, using an established reporting protocol and the Orchard Sports Injury Classification System. Furthermore, all injuries and illnesses were followed up post-tournament in order to obtain final diagnoses and return to play dates. In conclusion, the results from this study confirm a high incidence and severity of injury within international rugby but the incidence, nature and inciting events for injuries in RWC 2019 were broadly similar to those reported previously for RWC 2007, 2011 and 2015.

## Figures and Tables

**Table 1:** Anthropometric data of players competing at RWC 2019

Playing position (n)	Age (years)	Stature (cm)	Body mass (kg)
**Backs (n=284)**	27.3 (3.5)	182.6 (6.0)	91.2 (9.1)
Halves (n=101)	27.7 (3.6)	179.0 (5.9)	85.1 (7.5)
Inside backs (n=76)	27.7 (3.2)	185.5 (4.5)	97.5 (7.0)
Outside backs (n=107)	26.7 (3.6)	183.9 (5.4)	92.6 (8.1)
**Forwards (n=362)**	27.3 (3.8)	188.3 (7.0)	111.8 (9.2)
Front row (n=169)	27.2 (3.9)	183.7 (4.5)	114.4 (9.1)
Second row (n=80)	28.0 (3.6)	197.3 (4.6)	114.8 (6.6)
Back row (n=113)	27.1 (3.7)	188.9 (5.0)	105.8 (7.9)
**All players (n=646)**	27.3 (3.7)	185.8 (7.2)	102.8 (13.7)
**p value** *	1.000	<0.001	<0.001

Data are expressed as Mean (SD).

* indicates Backs vs Forwards RWC, Rugby World Cup; n, number of players

**Table 2:** Incidences and mean and median severities of injuries sustained by backs and forwards during matches and training

Activity / Playing position (n)	Incidence Injuries /1 000 player-hours (95% CI)	Severity Days (95% CI)	

		Mean	Median
**Match injuries (n)**			
**Backs (n=79)**	94.0 (75.4 – 117.3)	28.5 (17.2 – 39.8)	8 (6 – 11)
Halves (n=27)	112.5 (77.1 – 164.0)	24.6 (12.6 – 36.6)	7 (6 - 36)
Inside backs (n=20)	83.3 (53.8 – 129.2)	55.6 (17.1 – 94.0)	12 (8 – 67)
Outside backs (n=32)	88.9 (62.9 – 125.7)	14.9 (7.9 – 21.9)	6.5 (5 – 10)
**Forwards (n=64)**	66.7 (52.2 – 85.2)	29.4 (15.3 – 43.4)	9 (6 – 15)
Front row (n=29)	80.6 (56.0 – 115.9)	18.6 (9.4 – 27.7)	8 (4 – 23)
Second row (n=8)	33.3 (16.7 – 66.7)	39.0 (0 – 84.0)	14 (4 – 197)
Back row (n=27)	75.0 (51.4 – 109.4)	38.1 (9.1 – 67.1)	9 (5 – 28)
**All players (n=143)**	79.4 (67.4 – 93.6)	28.9 (20.0 – 37.7)	6 (3 – 13)
**p-value** *	0.041	0.920	0.992
**Training injuries (n)**			
**Backs (n=10)**	1.4 (0.8 – 2.6)	17.0 (0 – 42.3)	5.5 (2 – 15)
**Forwards (n=15)**	1.7 (1.0 – 2.7)	13.3 (6.5 – 20.1)	7 (2 – 27)
**All players (n=25)**	1.5 (1.0 – 2.3)	14.8 (4.1 – 25.5)	6 (3 – 13)
**p-value** *	0.674	0.779	0.978

* indicates Backs vs Forwards

n, number of injuries; CI, confidence interval

**Table 3:** Match injuries as a function of injury location and playing position

Injury location	% Proportion of injuries (95% CI)		

	Backs	Forwards	All players
**Head/neck injuries**	25.3 (15.7 – 34.9)	28.1 (17.1 – 39.1)	26.6 (19.3 – 33.8)
Head/face	22.8 (13.5 – 32.0)	21.9 (11.7 – 32.0)	22.4 (15.5 – 29.2)
Neck/cervical spine	2.5 (0 – 6.0)	6.3 (0.3 – 12.2)	4.2 (0.9 – 7.5)
**Upper limb injuries**	16.5 (8.3 – 24.6)	20.3 (10.5 – 30.2)	18.2 (11.9 – 24.5)
Shoulder/clavicle	6.3 (1.0 – 11.7)	9.4 (2.2 – 16.5)	7.7 (3.3 – 12.1)
Upper arm	0.0	1.6 (0 – 4.6)	0.7 (0 – 2.1)
Elbow	0.0	4.7 (0 – 9.9)	2.1 (0 – 4.4)
Forearm	1.3 (0 – 3.7)	1.6 (0 – 4.6)	1.4 (0 – 3.3)
Wrist/hand/fingers	8.9 (2.6 – 15.1)	3.1 (0 – 7.4)	6.3 (2.3 – 10.3)
**Trunk injuries**	7.6 (1.8 – 13.4)	9.4 (2.2 – 16.5)	8.4 (3.8 – 12.9)
Upper back/sternum/ribs	3.8 (0 – 8.0)	4.7 (0 – 9.9)	4.2 (0.9 – 7.5)
Abdomen	0.0	0.0	0.0
Lower back/pelvis	3.8 (0 – 8.0)	4.7 (0 – 9.9)	4.2 (0.9 – 7.5)
**Lower limb injuries**	50.6 (39.6 – 61.7)	42.2 (30.1 – 54.3)	46.9 (38.7 – 55.0)
Hip/groin	1.3 (0 – 3.7)	0.0	0.7 (0 – 2.1)
Anterior thigh	10.1 (3.5 – 16.8)	3.1 (0 – 7.4)	7.0 (2.8 – 11.2)
Posterior thigh	13.9 (6.3 – 21.6)	10.9 (3.3 – 18.6)	12.6 (7.2 – 18.0)
Knee	8.9 (2.6 – 15.1)	15.6 (6.7 – 24.5)	11.9 (6.6 – 17.2)
Lower leg/Achilles	3.8 (0 – 8.0)	6.3 (0.3 – 12.2)	4.9 (1.4 – 8.4)
Ankle	8.9 (2.6 – 15.1)	6.3 (0.3 – 12.2)	7.7 (3.3 – 12.1)
Foot/toe	3.8 (0 – 8.0)	0.0	2.1 (0 – 4.4)

CI, confidence interval

**Table 4:** Match injuries as a function of injury type and playing position

Injury type	% Proportion of injuries (95% CI)		

	Backs	Forwards	All players
**Bone injuries**	10.1 (3.5 – 16.8)	3.1 (0 – 7.4)	7.0 (2.8 – 11.2)
Fracture	10.1 (3.5 – 16.8)	3.1 (0 – 7.4)	7.0 (2.8 – 11.2)
Other bone injury	0.0	0.0	0.0
**C/PNS injuries**	15.2 (7.3 – 23.1)	20.3 (10.5 – 30.2)	17.5 (11.3 – 23.7)
Concussion	15.2 (7.3 – 23.1)	15.6 (6.7 – 24.5)	15.4 (9.5 – 21.3)
Nerve injury	0.0	4.7 (0 – 9.9)	2.1 (0 – 4.4)
**Joint (non-bone) injuries**	27.8 (18.0 – 37.7)	31.3 (19.9 – 42.6)	29.4 (21.9 – 36.8)
Dislocation/subluxation	3.8 (0 – 8.0)	1.6 (0 – 4.6)	2.8 (0.1 – 5.5)
Lesion meniscus/disc	5.1 (0.2 – 9.9)	4.7 (0 – 9.9)	4.9 (1.4 – 8.4)
Sprain/ligament	19.0 (10.3 – 27.6)	25.0 (14.4 – 35.6)	21.7 (14.9 – 28.4)
**Muscle/tendon injuries**	38.0 (27.3 – 48.7)	34.4 (22.7 – 46.0)	36.4 (28.5 – 44.2)
Haematoma/bruise	13.9 (6.3 – 21.6)	12.5 (4.4 – 20.6)	13.3 (7.7 – 18.9)
Muscle strain/cramp	20.3 (11.4 – 29.1)	20.3 (10.5 – 30.2)	20.3 (13.7 – 26.9)
Tendon injuries	3.8 (0 – 8.0)	1.6 (0 – 4.6)	2.8 (0.1 – 5.5)
**Skin injuries**	1.3 (0 – 3.7)	4.7 (0 – 9.9)	2.8 (0.1 – 5.5)
Abrasion	0.0	0.0	0.0
Laceration	1.3 (0 – 3.7)	4.7 (0 – 9.9)	2.8 (0.1 – 5.5)
**Pain (undiagnosed)**	3.8 (0 – 8.0)	3.1 (0 – 7.4)	3.5 (0.5 – 6.5)
**Other injuries**	3.8 (0 – 8.0)	3.1 (0 – 7.4)	3.5 (0.5 – 6.5)

C/PNS, central and peripheral nervous system; CI, confidence interval

**Table 5:** The most common match injuries and the match injuries causing most days absence

Most common injuries				Injuries causing most days absence			

Injury	n	%	Days absence	Injury	Days absence	%	n
Concussion	22	15.4	363	Knee ligament	935	22.6	8
Hamstring strain	14	9.8	467	Knee cartilage injury	495	12.0	5
Knee ligament	8	5.6	935	Hamstring strain	467	11.3	14
Ankle ligament	7	4.9	95	Concussion	363	8.8	22
Thigh haematoma	7	4.9	43	Shoulder dislocation	316	7.7	2
Knee cartilage injury	5	3.5	495	Calf muscle strain	194	4.7	4
All injuries	143		4 130	All injuries	4 130		143

n, number of injuries; %, proportion of all injuries

**Table 6:** Match injuries sustained as a function of playing position and match activity

Match activity	% Proportion of injuries (95% CI)		

	Backs	Forwards	All players
Tackling	29.3 (19.0 – 39.6)	27.9 (16.6 – 39.1)	28.7 (21.1 – 36.3)
Tackled	18.7 (9.8 – 27.5)	19.7 (9.7 – 29.6)	19.1 (12.5 – 25.7)
Running	21.3 (12.1 – 30.6)	11.5 (3.5 – 19.5)	16.9 (10.6 – 23.2)
Collision*	18.7 (9.8 – 27.5)	14.8 (5.9 – 23.7)	16.9 (10.6 – 23.2)
Ruck	4.0 (0 – 8.4)	6.6 (0.3 – 12.8)	5.1 (1.4 – 8.9)
Scrum	0.0	8.2 (1.3 – 15.1)	3.7 (0.5 – 6.8)
Maul	0.0	4.9 (0 – 10.3)	2.2 (0 – 4.7)
Kicking	2.7 (0 – 6.3)	0.0	1.5 (0 – 3.5)
Lineout	0.0	0.0	0.0
Other	5.3 (0.2 – 10.4)	6.6 (0.3 – 12.8)	5.9 (1.9 – 9.8)

* accidental and non-accidental collisions; CI, confidence interval
